# Clinical Characteristics and Outcomes of Clostridioides difficile Infection in Cancer Patients From a Tertiary Care Hospital

**DOI:** 10.7759/cureus.77616

**Published:** 2025-01-18

**Authors:** Muhammad Arslan, Muhammad Usman Shabbir, Umer Farooq, Baryah Bilal, Salma Abbas, Nahel Chaudhry, Muhammad Qasim, Summiya Nizamuddin

**Affiliations:** 1 Infectious Diseases, Shaukat Khanum Memorial Cancer Hospital and Research Centre, Lahore, PAK; 2 Internal Medicine, Shaukat Khanum Memorial Cancer Hospital and Research Centre, Lahore, PAK; 3 Medicine, Shaukat Khanum Memorial Cancer Hospital and Research Centre, Lahore, PAK; 4 Oncology, Shaukat Khanum Memorial Cancer Hospital and Research Centre, Lahore, PAK; 5 Microbiology, Shaukat Khanum Memorial Cancer Hospital and Research Centre, Lahore, PAK

**Keywords:** antibiotics, clostridioides difficile, hematological malignancy, risk factors, solid organ malignancy

## Abstract

Objective

The objective of this study is to investigate the predisposing factors, disease course, potential complications, role of primary prophylaxis, and overall clinical outcomes of *Clostridioides difficile* infection (CDI) in cancer patients.

Methods

The study was conducted at Shaukat Khanum Memorial Cancer Hospital and Research Centre, Lahore, Pakistan. We analyzed the medical records of cancer patients diagnosed with CDI from July 2015 to July 2024 and collected data about demographic characteristics, clinical presentation, predisposing factors, treatment, complications, and mortality rates. We used SPSS version 25 (IBM Corp., Armonk, NY) for data analysis.

Results

Out of 61 patients, 55.7% (n=34) were men, and most of the patients belonged to the age group of 41-65 years (49.1%; n=30). Of the patients, 34.4% (n=21) had underlying hematological malignancy, while the majority of patients (63.9%; n=39) had underlying solid organ malignancy. A total of 45.9% (n=28) of patients had mild severity, whereas 16.3% (n=10) and 6.55% (n=4) were at severe and fulminant stages of CDI, respectively. The creatinine levels of 80.3% (n=49) of patients were less than 1.5 mg/dL. We also observed the prior antimicrobial use, previous hospitalization within the last four weeks, recent chemotherapy, and use of proton pump inhibitors (PPIs)/H2 antagonists in the past four weeks as predisposing factors in 78.6% (n=48), 72.1% (n=44), 55.7% (n=34), and 75.4% (n=46) of patients, respectively. A greater proportion of patients (68.8%; n=42) had hospital/ICU stays of less than 15 days. Of the patients, 29.6% (n=18) had comorbid conditions such as diabetes mellitus (DM), chronic kidney disease (CKD), hypertension (HTN), ischemic heart disease (IHD), hepatitis, and atrial fibrillation. Oral vancomycin was administered as the primary treatment in 78.6% (n=48) of patients. We noted the resolution of symptoms in 91.8% (n=56) of patients, while 83.6% (n=51) of patients developed no complications. Additionally, the radiological findings of the patients were negative for toxic megacolon. Moreover, 4.91% (n=3) of patients had recurrent infections, whereas all-cause 30-day mortality was 13.1% (n=8). The mortality rate was higher in patients with solid organ tumors (17.9%; n=7) as compared to those having hematological malignancy (4.76%; n=1). Regression analysis showed that recent chemotherapy had an odds ratio (OR) of 11.550 (95% confidence interval {CI}: 1.332-100.9; p=0.998).

Conclusion

Cancer patients, especially those with solid tumors presenting with symptoms suggestive of CDI and prior chemotherapy exposure, need careful evaluation and preemptive treatment as CDI-related mortality is higher in cancer patients. Early diagnosis and treatment in this population can be lifesaving. Moreover, all cancer patients should receive CDI prophylaxis when indicated.

## Introduction

*Clostridioides difficile* (CD) is a Gram-positive, spore-producing bacillus frequently found in the human gastrointestinal tract, coexisting with a wide variety of anaerobic microbes. Its spore-forming ability allows it to survive for extended periods outside a host [1]. Over recent decades, CD has emerged as the primary pathogen responsible for healthcare-associated colitis, largely driven by the spread of highly virulent, epidemic strains [2]. The incidence and severity of *Clostridioides difficile* infection (CDI) depend on specific *C. difficile* strains, such as ribotypes 002, 018, 027, 56, 078-126, and 244, that are linked to antimicrobial resistance and higher risks of severe infection and relapse, making CD a significant concern in both healthcare and community settings [3,4]. The infection primarily affects those with compromised immune systems or disrupted gut microbiota, often leading to symptoms ranging from mild diarrhea to severe colitis and even life-threatening complications [5].

Cancer stands as the second leading cause of death worldwide, after cardiovascular diseases. In recent years, survival rates for cancer patients have improved significantly due to the introduction of targeted therapies along with established conventional treatments. While cancer progression itself heavily influences patient outcomes, infections remain a critical factor, contributing to notable morbidity and mortality among immunocompromised individuals. Nucleic acid amplification tests (NAATs) are highly sensitive for diagnosing CDI, but they cannot confirm active toxin production [4,6].

Cancer patients are at a heightened risk for CDI, especially in tertiary care settings. CDI risk is strongly linked to antibiotic use, which disrupts the gut microbiome, facilitating *C. difficile* colonization. Up to 15% of hospitalized patients may become colonized, as it has a tendency to spread from both symptomatic patients and carriers without symptoms [4]. Cancer patients are particularly susceptible due to their weakened immune systems, regular exposure to antibiotics especially within the last four weeks, the overcrowding of hospital wards, and extended hospital stays. Additional risk factors include chemotherapy, radiation therapy, proton pump inhibitors (PPIs), recent surgeries, and advanced age, which increase vulnerability to severe CDI and its complications [5,7].

The clinical outcomes of CDI in cancer patients vary, with an elevated risk of severe infection, recurrence, and, in some cases, increased mortality. Advanced age, coupled with comorbidities such as diabetes or chronic kidney disease (CKD), compounds this risk, leading to higher rates of severe infections, recurrence, and mortality [8]. Severe gastrointestinal involvement such as paralytic ileus and toxic megacolon indicates a fulminant life-threatening condition characterized by extreme colon dilation, inflammation, systemic toxicity, and sepsis leading to shock. Other complications include pseudomembranous colitis, severe dehydration, and chemotherapy-induced mucositis, which may further weaken their immune system and delay cancer treatments [9,10].

The primary treatment for CDI involves antibiotics aimed at eradicating* C. difficile *bacteria while preserving beneficial gut flora. Treatment options typically include fidaxomicin and vancomycin, two first-line agents with specific roles in managing CDI [11]. Treatment guidelines often emphasize the use of vancomycin over metronidazole for older patients and those with high comorbidity scores, as vancomycin is more effective in severe cases and has been associated with lower recurrence rates. In particular, oral vancomycin is preferred for moderate-to-severe CDI due to its targeted effect on the gastrointestinal tract, where CDI infection occurs. Evidence also suggests using fidaxomicin for recurrent CDI, preventing one additional recurrence at four weeks for every 10 patients treated [9,12].

In light of these risks, close monitoring and early intervention are critical for cancer patients with CDI. Given the complexity of treating CDI in this immunocompromised population, a multidisciplinary approach involving oncologists, infectious disease specialists, and gastroenterologists can be essential to outcomes and minimizing the risk of severe complications or death [13].

Despite advances in the management of CDI, there remains a controversy in the existing data regarding its unique clinical characteristics, risk factors, and outcomes in cancer patients, who are especially vulnerable due to immunosuppression, frequent antibiotic use, and chemotherapy. Therefore, the aim of this study is to investigate the predisposing factors, disease course, potential complications, role of primary prophylaxis, and overall clinical outcomes of CDI in cancer patients.

## Materials and methods

We conducted a retrospective, single-center, non-interventional, observational study. We included cancer patients with CDI diagnosed by a positive NAAT on a stool between July 2015 and July 2024. The study was conducted at Shaukat Khanum Memorial Cancer Hospital and Research Centre, Lahore, Pakistan, and included pediatric and adult patients. The Institutional Review Board (IRB) of Shaukat Khanum Memorial Cancer Hospital and Research Centre approved the study protocol (EX-05-08-24-19) and waived informed consent requirements due to its retrospective and anonymous nature. For each patient, baseline demographic data, cancer diagnosis and category, laboratory findings, underlying risk factors, clinical characteristics, complications, and outcomes were collected from the electronic institutional medical record. Data sheets were prepared with anonymized information to protect patient confidentiality. To analyze the relationship between CDI outcomes and predisposing factors, patients with hematological malignancies were compared to those with solid organ tumors across key variables. Frequencies and percentages were calculated for demographic and categorical variables, and statistical analysis was conducted using SPSS version 25 (IBM Corp., Armonk, NY). Chi-square (χ²) tests assessed associations between outcome variables and risk factors, with significance set at a p-value of <0.05. Multivariate logistic regression analysis was performed to assess the association between demographic and clinical factors and the likelihood of all-cause 30-day mortality (yes/no).

The diagnosis of CDI required three criteria: a positive nucleic acid amplification test for *C. difficile* toxin, the presence of diarrhea, and clinical symptoms consistent with CDI, i.e., fever, abdominal pain, distension, or tenderness. Immunosuppressive drugs commonly used in the cancer population that predispose patients to CDI include corticosteroids, immunosuppressants, and chemotherapeutic agents. Analysis was limited to agents available in the hospital formulary, primarily corticosteroids and commonly used chemotherapy drugs.

*Clostridioides difficile* infection was defined as a confirmed positive stool test for *C. difficile* toxin at the time of ongoing symptoms. Recurrence is defined as *Clostridioides difficile* infection after three months. Fever was defined as a temperature of 38°C or higher, while diarrhea was classified as three or more unformed stools within 24 hours. The severity of the disease was categorized based on the symptoms i.e., mild (afebrile and diarrhea without systemic findings), moderate (fever, profuse diarrhea, and abdominal pain), severe (fever, profuse diarrhea, abdominal pain, distention and tenderness, leukocytosis, elevated creatinine levels, and pseudomembranous colitis), and fulminant (hypotension, shock, ileus, and toxic megacolon) [14].

## Results

**Figure 1:**
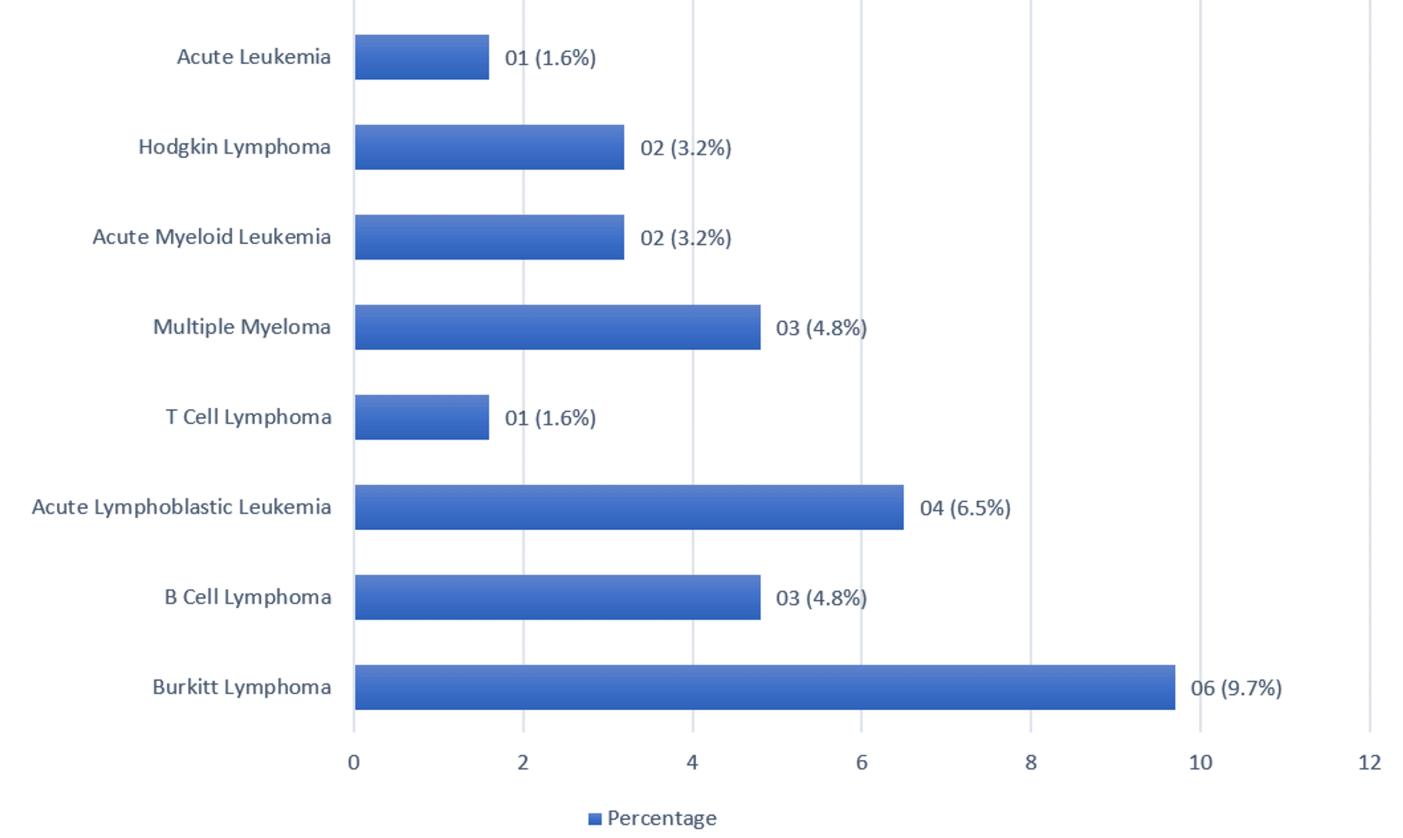
Percentage of various hematological malignancies in our study population (n=61)

**Figure 2:**
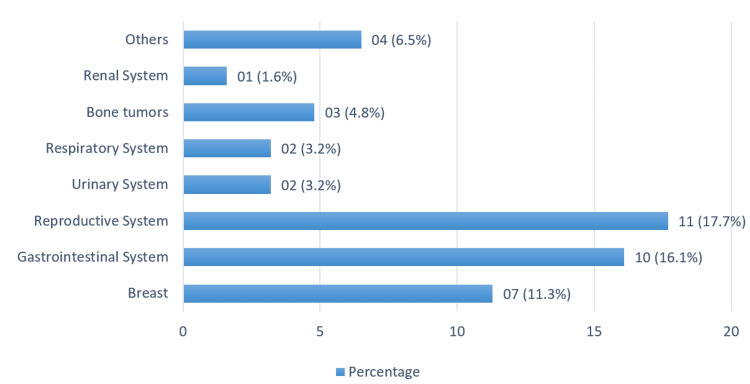
Percentage of various solid tumors in our study population (n=61)

A total of 13345 tests were performed, out of which 61 patients (0.45%) tested positive for *C. difficile* toxin. We studied and analyzed a total of 61 patients, of which 55.7% (n=34) were men, and most of the patients belonged to the age group of 41-65 years (49.1%; n=30). Of the patients, 34.4% (n=21) had underlying hematological malignancy, while the majority of patients (63.9%; n=39) had underlying solid organ malignancy. Regarding underlying risk factors, 55.7% (n=34) of patients had received recent chemotherapy, with 80.9% (n=17) in hematological malignancies and 33.3% (n=16) in solid organ tumors (Table 1). Within the hematological malignancies, those with Burkitt lymphoma were the most prevalent (Figure 1), whereas reproductive system tumors (ovarian carcinoma, cervical cancer, and prostate carcinoma) were the most common among solid organ malignancies (Figure 2). The percentages of various hematological malignancies and solid organ tumors in our study group are represented in Figure 1 and Figure 2.

**Table 1:** Demographic and clinical characteristics of CDI patients with outcomes The data has been represented in the form of percentages (%) and numbers (n). A p-value of less than 0.05 has been considered significant *A significant p-value -An empty cell CDI, *Clostridium difficile* infection; PPI, proton pump inhibitor

Clinical or demographic characteristics		Total percentage of all patients (n)	Percentage in hematological malignancy (n)	Percentage in solid organ tumors (n)	Percentage in both hematological and solid tumor (n)			P-value (<0.05 is significant)
Demographics	100%; n=61		34.4%; n=21	63.9%; n=39	1.63%; n=1		-	
Gender								
Male		55.7%; n=34	66.6%; n=14	48.7%; n=19	100%; n=1	0.113		
Female		44.3%; n=27	33.3%; n=7	51.3%; n=20	0.00%; n=0			
Age								
<20		21.3%; n=13	52.4%; n=11	5.13%; n=2	0.00%; n=0	0.290		
20-40		22.9%; n=13	28.6%; n=6	17.9%; n=7	100%; n=1			
41-65		49.1%; n=30	19.0%; n=4	66.6%; n=26	0.00%; n=0			
>65		6.55%; n=4	0.00%; n=0	10.2%; n=4	0.00%; n=0			
Delay in testing (in days)								
0-7 days		90.1%; n=55	95.2%; n=20	61.8%; n=34	100%; n=1	0.001		
8-14 days		4.91%; n=3	4.76%; n=1	5.13%; n=2	0.00%; n=0			
15-21 days		4.91%; n=3	0.00%; n=0	10.2%; n=3	0.00%; n=0			
Laboratory investigations								
Hemoglobin (g/dL)								
5-10		77.1%; n=47	85.7%; n=18	74.3%; n=29	0.00%; n=0	0.110		
11-15		22.9%; n=14	14.3%; n=3	25.6%; n=10	100%; n=1	-		
WBC (10^9^/L)								
0-10		62.2%; n=38	76.2%; n=16	53.8%; n=21	100%; n=1	-		
10-20		34.4%; n=21	23.8%; n=5	41.0%; n=16	0.00%; n=0	0.409		
20-30		3.27%; n=2	0.00%; n=0	5.13%; n=2	0.00%; n=0	-		
Creatinine (mg/dL)								
≤1.5		80.3%; n=49	90.5%; n=19	74.4%; n=29	100%; n=1	0.021*		
>1.5		19.6%; n=12	9.50%; n=2	25.6%; n=10	0.00%; n=0	-		
Severity								
Mild		45.9%; n=27	38.1%; n=8	48.7%; n=19	100%; n=1	0.016*		
Moderate		31.1%; n=19	52.4%; n=11	20.5%; n=8	0.00%; n=0			
Severe		16.3%; n=10	9.50%; n=2	20.5%; n=8	0.00%; n=0			
Fulminant		6.55%; n=4	0.00%; n=0	10.3%; n=4	0.00%; n=0			
Predisposing/risk factors								
Comorbid conditions								
No		70.4%; n=43	76.2%; n=16	66.6%; n=26	100%; n=1	0.595		
Yes		29.6%; n=18	23.8%; n=5	33.3%; n=13	0.00%; n=0			
Antimicrobial use within the last four weeks								
No		21.3%; n=13	14.3%; n=3	23.1%; n=9	100%; n=1	0.170		
Yes		78.6%; n=48	85.7%; n=18	76.9%; n=30	0.00%; n=0			
Previous hospitalization within the last four weeks								
No		27.8%; n=17	19.1%; n=4	33.3%; n=13	0.00%; n=0	0.080		
Yes		72.1%; n=44	80.9%; n=17	66.6%; n=26	100%; n=1			
Duration of hospital/ICU stay								
<15 days		68.8%; n=42	61.9%; n=13	71.7%; n=28	100%; n=1	0.080		
15-30 days		26.2%; n=16	38.1%; n=8	20.5%; n=8	0.00%; n=0			
31-45 days		3.27%; n=2	0.00%; n=0	5.13%; n=2	0.00%; n=0			
>45 days		1.63%; n=1	0.00%; n=0	2.56%; n=1	0.00%; n=0			
Mechanical ventilation								
No		93.4%; n=57	100%; n=21	89.7%; n=35	100%; n=1	0.024*		
Yes		6.55%; n=4	0.00%; n=0	10.2%; n=4	0.00%; n=0			
Recent chemotherapy within the last four weeks								
No		44.2%; n=27	19.1%; n=4	66.6%; n=23	0.00%; n=0	0.008*		
Yes		55.7%; n=34	80.9%; n=17	33.3%; n=16	100%; n=1			
PPI/H2 antagonist use within the last four weeks								
No		24.5%; n=15	14.3%; n=3	28.2%; n=11	100%; n=1	0.048*		
Yes		75.4%; n=46	85.7%; n=18	71.8%; n=28	0.00%; n=0			
Treatment								
Oral vancomycin		78.6%; n=48	80.9%; n=17	76.9%; n=30	100%; n=1	0.529		
Metronidazole		6.56%; n=4	9.50%; n=2	5.13%; n=2	0.00%; n=0			
Combination of metronidazole and vancomycin		14.7%; n=9	9.50%; n=2	17.9%; n=7	0.00%; n=0			
Outcomes								
Resolution of symptoms								
No		8.19%; n=5	0.00%; n=0	12.8%; n=5	0.00%; n=0	0.006*		
Yes		91.8%; n=56	100%; n=21	87.2%; n=34	100%; n=1			
Complications								
No		83.6%; n=51	85.7%; n=18	82.1%; n=32	100%; n=1	0.048*		
Yes		16.4%; n=10	14.3%; n=3	17.9%; n=7	0.00%; n=0			
Radiology (toxic megacolon)								
No		100%; n=61	100%; n=21	100%; n=39	100%; n=1	-		
Yes		0.00%; n=0	0.00%; n=0	0.00%; n=0	0.00%; n=0			
Recurrence/readmission								
No		95.1%; n=58	90.5%; n=19	97.4%; n=38	100%; n=1	0.178		
Yes		4.91%; n=3	9.50%; n=2	2.56%; n=1	0.00%; n=0			
All-cause 30-day mortality								
No		86.8%; n=53	95.2%; n=20	82.1%; n=32	100%; n=1	0.013*		
Yes		13.1%; n=8	4.76%; n=1	17.9%; n=7	0.00%; n=0			

We also observed the prior antimicrobial use, previous hospitalization, recent chemotherapy, and use of proton pump inhibitors/H2 antagonists in the past four weeks as predisposing factors in 78.6% (n=48), 72.1% (n=44), 55.7% (n=34), and 75.4% (n=46) of patients, respectively. A greater proportion of patients (68.8%; n=42) had hospital/ICU stays of less than 15 days. Among patients with CDI, 29.6% (n=18) patients had comorbid conditions such as diabetes mellitus (DM), CKD, hypertension (HTN), ischemic heart disease (IHD), hepatitis, and atrial fibrillation. Additionally, treatment for CDI predominantly involved oral vancomycin administered to 78.6% (n=48) of patients. A smaller subset, 14.7% (n=9), required a combination therapy of vancomycin and metronidazole.

Out of 61 cases, 45.9% (n=28) had mild severity, whereas 16.3% (n=10) and 6.55% (n=4) were at severe and fulminant stages of CDI, respectively. Fulminant cases were reported only in patients with solid organ tumors (10.3%; n=4). We noted the resolution of symptoms in 91.8% (n=56) of patients, while 83.6% (n=51) of patients developed no complications. All the patients with hematological malignancies demonstrated complete resolution of symptoms (100%; n=21). Complications were documented only in 16.4% (n=10) of patients. It was also observed that the radiological findings of all the patients were negative for toxic megacolon. The laboratory investigations revealed creatinine levels of <1.3 mg/dL in the majority (80.8%; n=49) of cases; only 12 patients (19.6%) had a serum creatinine of >1.5 mg/dL. Moreover, creatinine levels were markedly elevated in patients with solid organ tumors (25.6%; n=10) as compared to those with hematological malignancy (9.50; n=2). A large proportion (77.1%; n=47) was anemic, with their hemoglobin levels ranging from 5 to 10 g/dL.

Moreover, recurrence was documented in 4.91% (n=3) of patients. Mortality analysis revealed a 30-day all-cause mortality rate of 13.1% (n=8) among the study group, with mortality higher among patients with solid organ malignancies in comparison to those with hematological malignancies (17.9% {n=7} in comparison to 4.76% {n=1}; p-value of 0.013). Out of eight mortality cases, 75% (n=6) were men, while 25% (n=2) were women (p-value of 0.113). According to our study, risk factors for CDI-related higher mortality in cancer patients include male gender, older age, previous hospitalization in the last four weeks, PPI use in the last four weeks, prior antibiotic use in the last four weeks, and the presence of underlying solid organ malignancy. The percentages and p-values of demographic and clinical characteristics and outcomes are given in Table 1.

We also conducted multivariate logistic regression to assess the association between demographic and clinical factors and the likelihood of all-cause 30-day mortality (yes/no). Independent variables included age, gender, the type of malignancy, PPI/H2 antagonist use within the last four weeks, mechanical ventilation, recent chemotherapy, and antibiotic use within the last four weeks. The Hosmer-Lemeshow test indicated a good fit for the model (χ²=2.927; p=0.939) as shown in Table 2. The Nagelkerke R^2^ was 0.456, suggesting that the model explained 45% of the variance in all-cause 30-day mortality status. Overall, the accuracy was 88.5% with 100% specificity and 12.5% sensitivity for mortality within 30 days.

**Table 2:** Hosmer-Lemeshow goodness of fit A p-value of >0.05 has been considered insignificant to predict that the model has an adequate fit df: degrees of freedom

Hosmer-Lemeshow test			
Step	Chi-square	df	Significance
1	2.927	8	0.939

The results of logistic regression analysis, including coefficients (B), odds ratio (OR), 95% confidence intervals (CIs) for OR, and p-values, are summarized in Table 3.

**Table 3:** Results of multivariate logistic regression A p-value of less than 0.05 has been considered significant PPI, proton pump inhibitor; CI, confidence interval

Variable	Coefficient (B)	Odds ratio (OR)	95% CI for OR	P-value
Malignancy	0.040	1.040	0.079-13.759	0.976
Age	-0.570	0.566	0.175-1.827	0.341
Gender	-0.244	0.784	0.099-6.209	0.817
Recent chemotherapy	2.447	11.550	1.332-100.9	0.998
Mechanical ventilation	-2.140	0.118	0.014-0.995	0.998
PPI/H2 antagonist use within the last four weeks	-1.287	0.276	0.008-0.9331	0.474
Antibiotic use within the last four weeks	0.298	1.348	0.039-46.652	0.869

PPI/H2 antagonist use and age were not significantly associated with mortality (p=0.474 and p=0.341, respectively). Likewise, the type of malignancy, gender, and prior antibiotic use within the last four weeks had wide confidence intervals and were not statistically significant. Recent chemotherapy had an odds ratio of 11.550 (95% CI: 1.332-100.9; p=0.998) However, the result was not statistically significant. Mechanical ventilation was associated with lower odds of mortality (OR=0.118, 95% CI: 0.014-0.995, and p=0.998), but the p-value was not statistically significant.

## Discussion

This study is the first in Pakistan to analyze the clinical characteristics and outcomes of CDI in cancer patients from a tertiary care hospital. While this adds valuable insight to a largely under-researched area, more extensive studies are needed to better understand CDI in this vulnerable population. Our findings revealed a low positivity rate of 0.45% (61 out of 13345 tests) despite conducting a substantial number of tests. This low rate may have influenced the overall statistical power and generalizability of the results, emphasizing the need for further large-scale studies.

The demographics in our study revealed a predominance of older age groups, especially those between 41 and 65 years (49.1%; n=30), highlighting that CDI in cancer patients is more common in older adults in our settings. Male patients made up 55.7% (n=34), suggesting a gender-based trend that may reflect the distribution of cancer types and associated treatments rather than a specific CDI predisposition. Similar findings have been observed in studies focusing on CDI in immunocompromised populations, where older age and gender distribution are prominent due to their influence on cancer incidence and treatment-related immune suppression [6,15].

In patients with CDI undergoing cancer treatment, identifying predisposing factors is critical for timely diagnosis and management. In our study, a significant finding was the association of CDI with recent chemotherapy exposure. Specifically, 55.7% (n=34) of patients had undergone recent chemotherapy, with 80.9% (n=17) in hematological malignancies and 33.3% (n=16) in solid tumors. This finding aligns with prior research indicating chemotherapy’s immunosuppressive effects, which increase susceptibility to CDI in cancer patients [16].

The findings of multivariate logistic regression highlighted that recent chemotherapy significantly increased the odds of 30-day all-cause mortality in cancer patients with CDI (OR: 11.550, 95% CI: 1.332-100.921, and p=0.998), highlighting it as a critical risk factor. In contrast, factors such as age, gender, recent antibiotic use, and mechanical ventilation demonstrated no statistical significance with mortality. A prior analysis of over 30 million cancer discharges in US community hospitals found that CDI was associated with significantly higher mortality rates (9.4% versus 7.5%) and longer median hospital stays (nine days versus four days) compared to patients without CDI [17]. A 2023 observational study assessed the performance of CDI severity scoring systems in cancer patients. The study identified neutropenia, male gender, elevated serum creatinine, and low albumin levels as significant risk factors for 30-day all-cause mortality [18]. A Swedish cohort study reported that individuals with CDI had a three- to fourfold increased mortality rate compared to matched controls, even after adjusting for chronic comorbidities [19].

The severity of CDI in cancer patients was a significant determinant of outcomes. Approximately 16.3% of patients presented with severe disease, while 6.55% developed fulminant CDI. Severe and fulminant CDI cases were associated with higher mortality rates (p=0.013), indicating the need for early recognition and aggressive management in at-risk populations. The predominance of severe cases in patients with solid tumors (20.5%; n=8) as compared to hematological malignancies (9.50%; n=2) and all the fulminant cases (10.3%; n=4) being exclusively reported in solid tumors emphasizes tumor type as a critical risk factor. Prior researches also support this potential tumor-type-specific susceptibility to severe CDI [4,20]. Wang et al. observed that patients with solid organ tumors represented a significant proportion of severe CDI cases compared to those with hematological malignancies. Their findings suggest that the type of cancer may influence the severity of CDI, potentially due to differences in host immune responses, treatment regimens, or other underlying factors associated with tumor biology [21].

Elevated leukocyte counts and serum creatinine levels were prominent markers of CDI severity in our study. Leukocytosis (WBC of >20×10⁹/L) was exclusively noted in severe cases, consistent with findings by Zhang et al. who highlighted leukocytosis as a key predictor of CDI severity and treatment failure. Similarly, elevated creatinine levels (>1.5 mg/dL) were observed in 19.6% (n=12) of our patients, especially in patients with solid organ tumors (25.6%; n=10) as compared to those with hematological malignancy (9.50; n=2), aligning with studies that identify renal impairment as a significant risk factor for severe CDI and poor outcomes [22]. Moreover, it was reported that leukocytosis and renal dysfunction are strong indicators of disease progression and adverse outcomes in CDI patients. These findings align with the IDSA-SHEA criteria, which define fulminant CDI as the presence of leukocytosis (WBC of ≥ 15000 cells/mL) or elevated serum creatinine levels (>1.5 mg/dL), among other clinical indicators. While the thresholds in our study differed slightly, the consistent association of higher leukocyte counts and serum creatinine levels with disease severity emphasizes their role as practical markers for assessing and managing CDI severity in vulnerable populations such as cancer patients [23,24].

Mechanical ventilation was another significant factor in our study, albeit affecting a smaller subset of patients (6.55%; n=4). Notably, all these patients had solid tumors, which may point to more advanced disease or complications necessitating intensive care support. Mechanical ventilation is associated with alterations in respiratory and gut microbiota, along with increased hospital-acquired infection risk, which can predispose patients to CDI. Our findings underscore the critical need for a multidisciplinary approach, combining infection control measures with a tailored care plan for high-risk cancer patients to mitigate CDI risk and improve outcomes in tertiary care hospitals [25,26].

Our study also revealed a noteworthy association between proton pump inhibitor (PPI) or H2 antagonist use and CDI occurrence, with 75.4% (n=46) of patients having received these medications within the last four weeks. The impact of PPIs on gut microbiota and gastric acid suppression is well-documented, potentially contributing to CDI by creating an environment conducive to pathogen overgrowth. The p-value (0.048) associated with PPI use suggests a statistically significant risk factor, emphasizing the need for the careful assessment of PPI indications in cancer patients. Prior studies have similarly linked PPIs to increased CDI risk, further supporting our findings and highlighting the importance of judicious PPI use in vulnerable patient populations [25-27].

Our study also underscored the impact of hospitalization-related risk factors on CDI occurrence. Patients with recent hospitalizations within four weeks accounted for 72.1% (n=44) of CDI cases, and 78.6% (n=48) had recent antimicrobial exposure. These observations underscore the role of the hospital environment and prior antibiotic exposure in CDI risk, consistent with established literature linking inpatient settings and antibiotic use with increased CDI incidence. Furthermore, a prolonged hospital stay of over 15 days was observed in 31.9% (n=19) of patients, a notable factor given that extended hospital exposure may lead to higher CDI rates due to longer contact with healthcare-associated pathogens [6,7,25,28].

Oral vancomycin was the primary treatment in this study, administered to 78.6% (n=48) of patients, as fidaxomicin was unavailable at our facility. Despite this limitation, symptom resolution was achieved in 91.8% (n=56) of cases, and the recurrence rate was low (4.91%, n=3), highlighting the efficacy of vancomycin in resource-limited settings but also highlighting the need for careful follow-up to prevent recurrent infections. This treatment approach is aligned with current IDSA guidelines, which suggest fidaxomicin along with vancomycin (as an alternative) as a first-line treatment for CDI in immunocompromised patients [9,12].

Regarding clinical outcomes, all-cause mortality within 30 days was observed in 13.1% (n=8) of patients, predominantly in those with solid tumors (17.9%; n=7). This contrasts with earlier studies where hematological malignancy patients typically exhibited higher CDI-related mortality, possibly due to different immune response mechanisms or variations in underlying cancer therapies [6,29]. Despite effective treatment, complications occurred in 16.4% (n=10) of cases, particularly among patients with solid tumors. The higher complication rate among solid tumor patients could reflect the deeper immunosuppression associated with their malignancies and treatments [9,30].

The limitations of our study include its retrospective design, single-center scope, and modest sample size, which limited statistical power and the ability to detect significant associations in regression analysis. Furthermore, the lack of prospective data collection limits our ability to assess causative temporal relationships and patient histories in real time. Additionally, the low positivity rate (0.45%) further reduced dataset variability. Nevertheless, our findings contribute valuable insights into CDI characteristics and outcomes in cancer patients. Future studies should aim for multicenter, large-scale analyses to confirm these associations and explore potential interventions tailored to at-risk cancer populations. This could pave the way for targeted CDI prophylaxis and treatment protocols that better address the needs of cancer patients in tertiary care environments.

## Conclusions

The findings of our research concluded that CDI presents a higher risk for cancer patients with solid organ tumors, compared to patients with hematological malignancies, particularly those who are recently exposed to antibiotics and had prior PPI use, recent chemotherapy within the last four weeks, and use of mechanical ventilation. Moreover, the study identified leukocytosis and elevated serum creatinine as potential indicators of severity. We suggest that all patients with symptoms suggestive of gastrointestinal infection should be cautiously evaluated for *C. difficile* infection in addition to adherence to the hygiene measures and not overcrowding the wards. Additionally, we suggest that future studies may target prospective, multicenter, and large-scale analyses to confirm these associations that can explore potential interventions tailored to at-risk cancer populations. 
